# 

**Published:** 1908-09-05

**Authors:** 


### CONTENTS.

| | PAGE |
|---|---|
| **Medicine as a Profession** | 589 |
| **The Cost of Medical Education** | 591 |
| **Lectures and Examinations** | 592 |
| **Medical Education in London** | 593 |
| **The Medical Curriculum—** | |
| Qualification and Registration | 594 |
| The English Conjoint Course | 594 |
| The Cambridge Course | 595 |
| The London University Course | 596 |
| The Fellowship | 596 |
| Higher Qualifications for Practitioners | 597 |
| **Notes on Medical Schools and Colleges—** | |
| The Choice of a Medical School | 603 |
| The Medical Schools of the United Kingdom | 604 |
| **The Public Services—** | |
| The Naval and Military Services as a Career | 598 |
| Details of His Majesty's Services | 599 |
| Tropical Medicine | 600 |
| The Public Health Service as a Career for Medical Men | 601 |
| Public Health Diplomas | 602 |
| **Graduate Medical Study—** | |
| Some General Considerations | 609 |
| London Post-Graduate Institutions | 610 |
| Opportunities for Graduate Study in Special Hospitals | 612 |
| Post-Graduate Study in Scotland and Ireland | 613 |
| Graduate Study on the Continent | 613 |
| **The Medical Library—** | |
| The Selection of Medical Books | 614 |
| Handbooks for Senior Students and Practitioners | 615 |

